# Capillary refill time: testing microcirculatory responses to maintain blood flow

**DOI:** 10.1186/s40635-025-00749-5

**Published:** 2025-07-31

**Authors:** Gustavo A. Ospina-Tascón, Eduardo Kattan, Jan Bakker, Glenn Hernández

**Affiliations:** 1 Department of Intensive Care Medicine, Fundación Valle del Lili, Cali, Colombia; 2 Translational Research Laboratory in Critical Care Medicine (TransLab-CCM), Universidad Icesi, Cali, Colombia; 3 Departamento de Medicina Intensiva, Facultad de Medicina, Pontificia Universidad Católica de Chile, Santiago, Chile; 4 Department of Intensive Care Adults, Erasmus MC University Medical Center, Rotterdam, Netherlands

To the Editor,

During lower body negative pressure (LBNP) challenge eliciting significant decreases in arterial pressure in healthy volunteers, Gustavsson et al. [1] observed a significant improvement in capillary refill time (CRT) at maximum provocation in 2/3 of the participants with an overall shortage of 0.2 s (a clinically non-significant value). As expected, reduction of venous return was accompanied by drop in cardiac output and compensatory increase of vascular resistances. Results from this experiment are interesting and fully illustrative of preserved physiological response of the cardiovascular system to an acute decrease in venous return in healthy people. Consequently, we disagree when they conclude that “CRT paradoxically decreases” during this hemodynamic challenge and suggest that these results “challenge the reliability of CRT as indicator of shock”.

LBNP is a well-known experimental method to reproduce hemodynamic and neurohormonal changes in response to central hypovolemia provoked by blood shift from upper to lower body compartments without orthostatic loading in conscious humans. Changes occurred during LBNP recreates initial responses to hypovolemia such as those observed during pre-shock stages of hemorrhage, but do not reproduce the shock phenomenon itself, which is defined as a life-threatening acute circulatory failure in which circulation is unable to deliver sufficient oxygen to meet tissue demands resulting in cellular dysfunction. Thus, although LBNP challenge induces hypotension and might reduce oxygen delivery to tissues, these changes are usually tolerated by cells during relatively short periods, and do not reduce tissue oxygen consumption significantly [2].

Organ blood flow remains almost constant in a wide range of perfusion pressures as consequence of tightly regulated mechanisms including the adaptation of diameters of first-order to terminal arterioles in response to variations in transmural pressures (i.e., myogenic responses), the reaction of vascular smooth muscle to cell and endothelial retrograde signals, and the vascular sympathetic influx. Thus, when transmural pressures suddenly drop, vasodilation occurs as consequence of the myogenic response [3] while central hypovolemia elicits baroreflex-mediated increase of muscle sympathetic nerve activity, both aiming to restore regional and organ blood flows. Preservation or abolition of these mechanosensitive and autonomic mechanisms might be recreated by inducing reactive hyperemia or any ischemia–reperfusion challenge, such as CRT. Consequently, shortening of CRT some minutes after starting LBNP challenge reflects preservation of microcirculatory vasoreactivity mechanisms to sudden hypotension rather than a “paradoxical response” as signaled by Gustavsson et al. [1], although admittedly, the huge variation of baseline CRT values (some of them grossly abnormal) overcloud the interpretation of their results.

CRT should be understood as a dynamic test evaluating the preservation or disruption of normal responses of microcirculation to maintain blood flow after a short ischemic challenge. Consequently, reliability of CRT should not be challenged by demonstrating perfectly normal responses of microcirculatory vasoreactivity to elicited sudden hypotension (but not shock) in healthy people. Conversely, a persistently abnormal CRT after initial fluid resuscitation identifies more severe cases of already diagnosed septic shock [4] and potential need for intervention, while its normalization might allow a safe stopping of resuscitation efforts [5].

## Data Availability

Authors declare no additional data or materials were used to draft this letter.
